# Supplementary figures and images for: TK1 expression influences pathogenicity by cell cycle progression, cellular migration, and cellular survival in HCC 1806 breast cancer cells
Source: PLoS One. 2023 Nov 30;18(11):e0293128. doi: 10.1371/journal.pone.0293128 (PMC10688958; doi:10.1371/journal.pone.0293128)

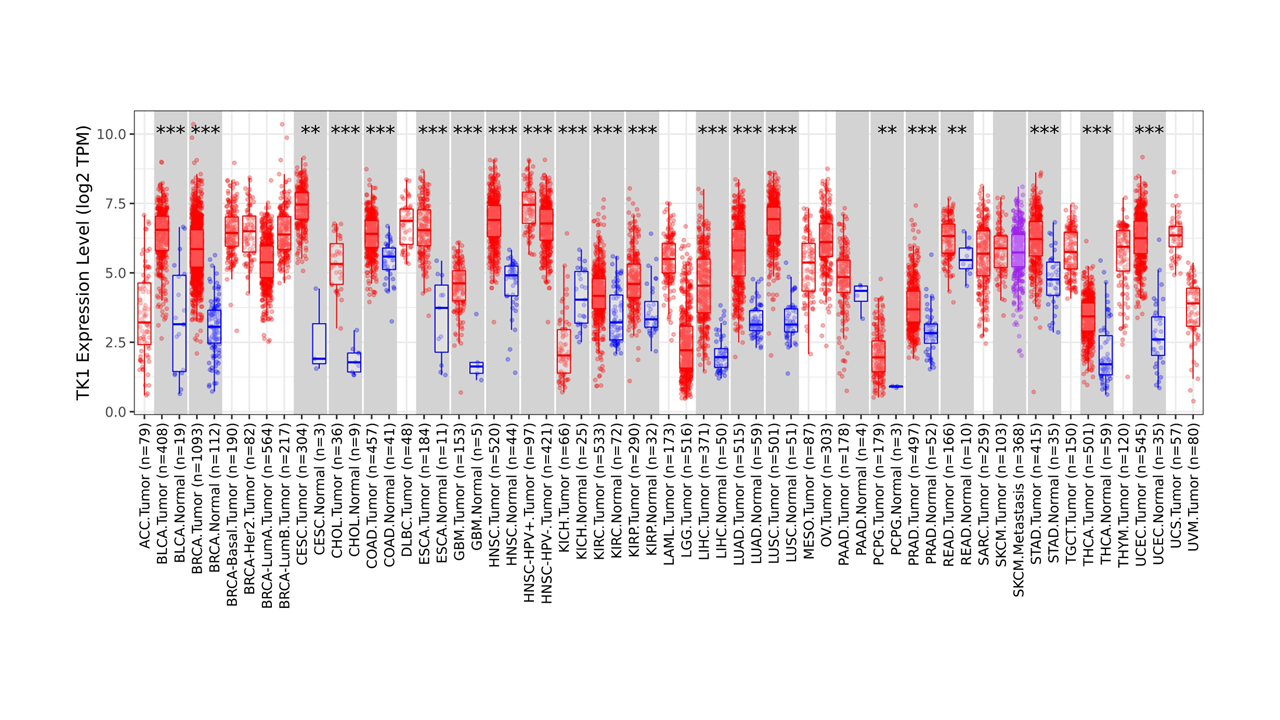

Supplement: S1 Fig — RNA TK1 differential expression was examined between adjacent normal and tumor tissue. RNA-seq data was extracted from The Cancer Genome Atlas (TCGA) for 39 different cancer types. TK1 RNA expression was significantly higher in cancer tissue when compared to adjacent normal samples for 20 of the 39 cancer types evaluated. Wilcoxon test was used to test significance and is shown by the number of stars (**: p-value <0.01; ***: p-value <0.001). Columns in gray denote when normal data are available. (TIF) [file pone.0293128.s001.tif]

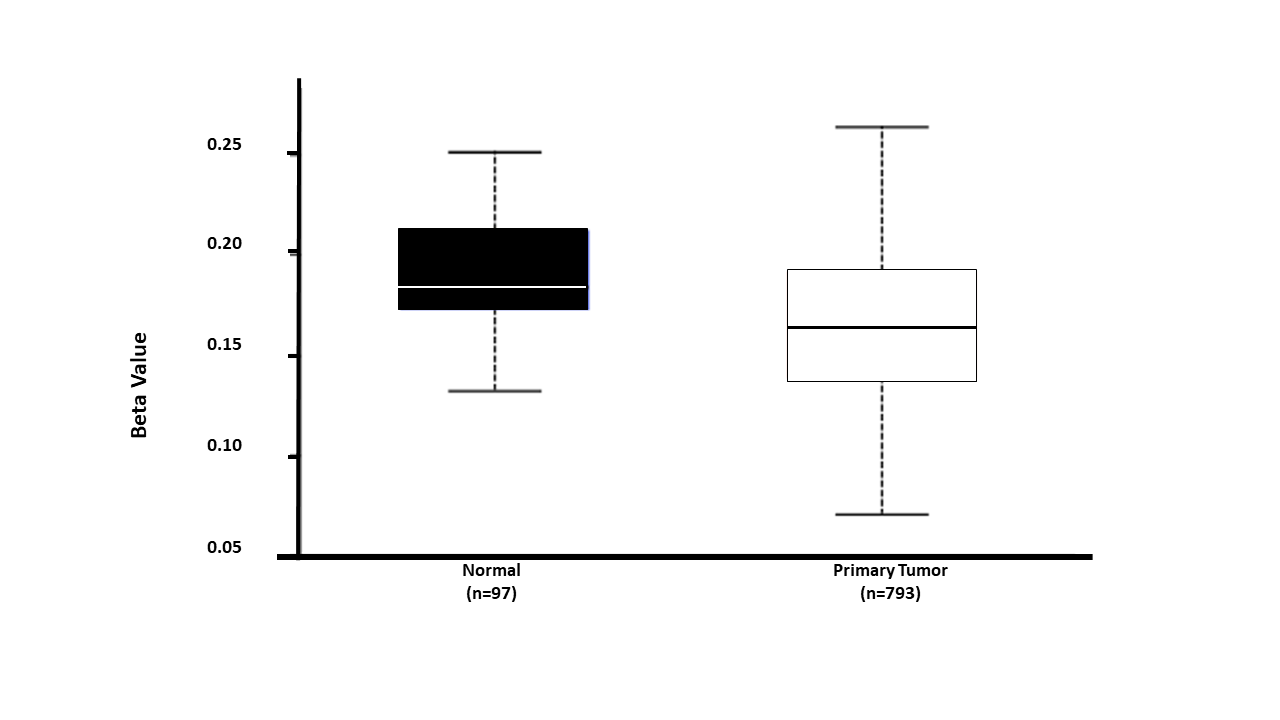

Supplement: S2 Fig — The data shown is described using a box and whisker plot and shows the average β value. The beta value indicates level of DNA methylation ranging from 0 (unmethylated) to 1 (fully methylated). The difference between the tumor and normal samples was estimated by a student’s t-test in which unequal variance was considered. Normal patient samples showed a higher level of methylation in the TK1 promoter when compared to primary tumor samples (p = 1.11 x 10^–16^). (TIF) [file pone.0293128.s002.tif]

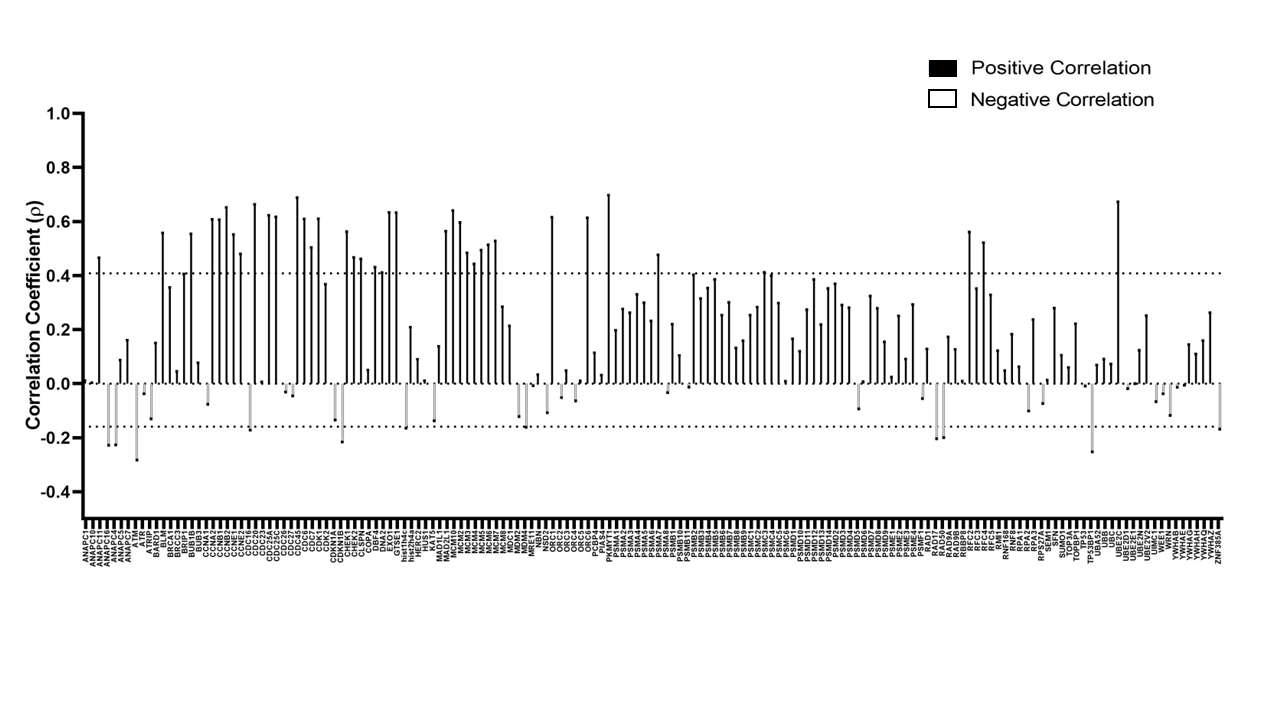

Supplement: S3 Fig — Significant Spearman correlations (p ≤ 0.05) are shown for positive (n = 124) and negative (n = 37) coefficients. Stronger correlations extend above or below two standard deviations that are denoted by horizontal asymptotes at 0.408 and -0.159. (TIF) [file pone.0293128.s003.tif]

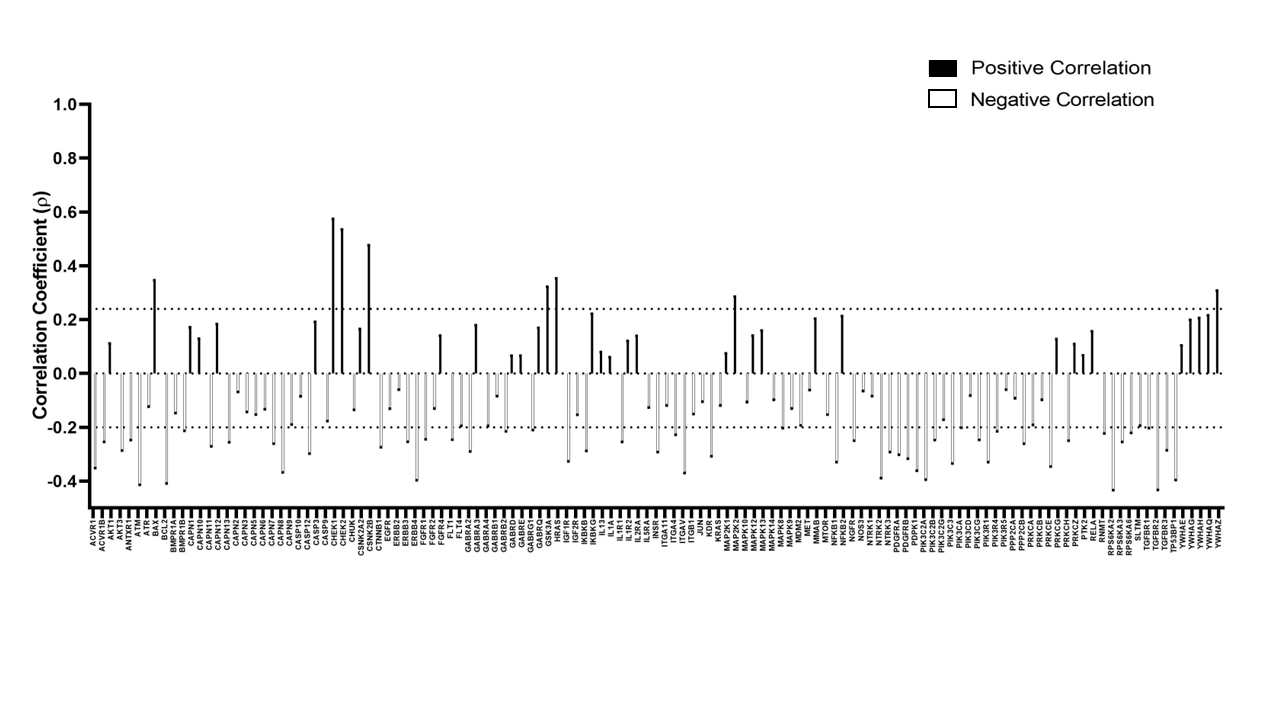

Supplement: S4 Fig — Significant Spearman correlations (p ≤ 0.05) are shown for positive (n = 37) and negative (n = 90) coefficients. Stronger correlations extend above or below two standard deviations that are denoted by horizontal asymptotes at 0.24 and -0.20. (TIF) [file pone.0293128.s004.tif]

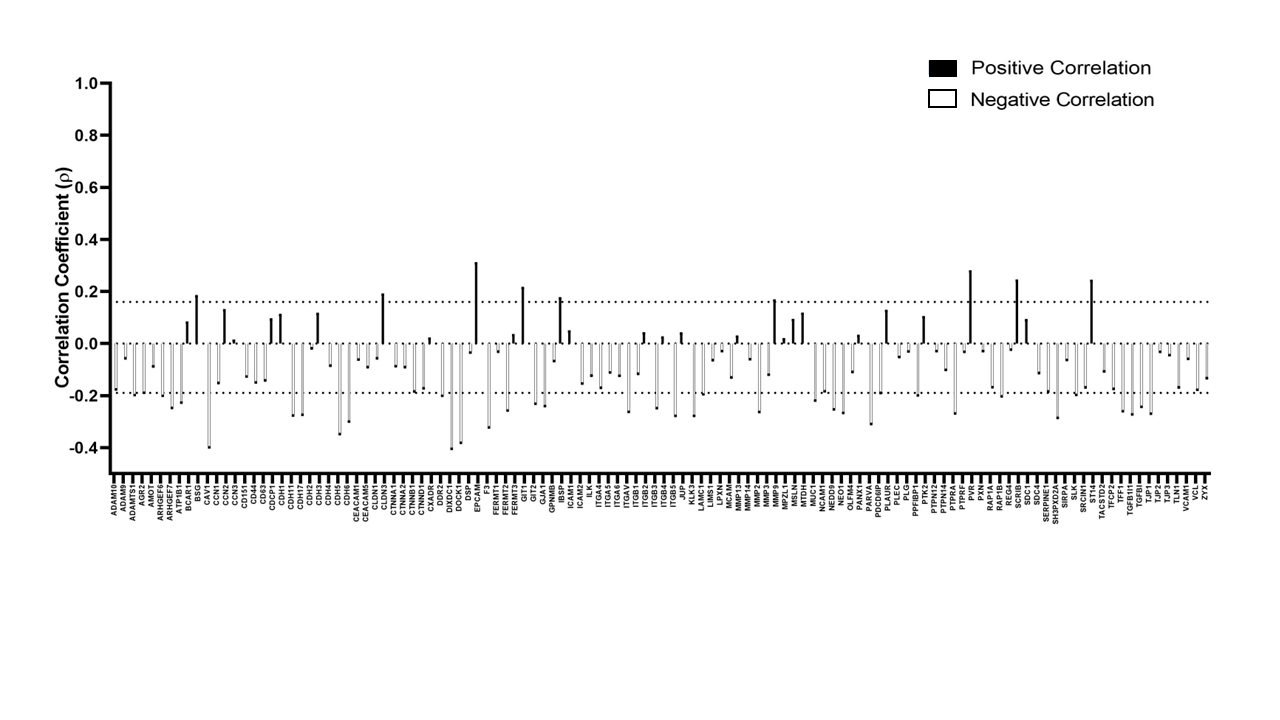

Supplement: S5 Fig — Significant Spearman correlations (p ≤ 0.05) are shown for positive (n = 29) and negative (n = 89) coefficients. Stronger correlations extend above or below two standard deviations that are denoted by horizontal asymptotes at 0.16 and -0.19. (TIF) [file pone.0293128.s005.tif]

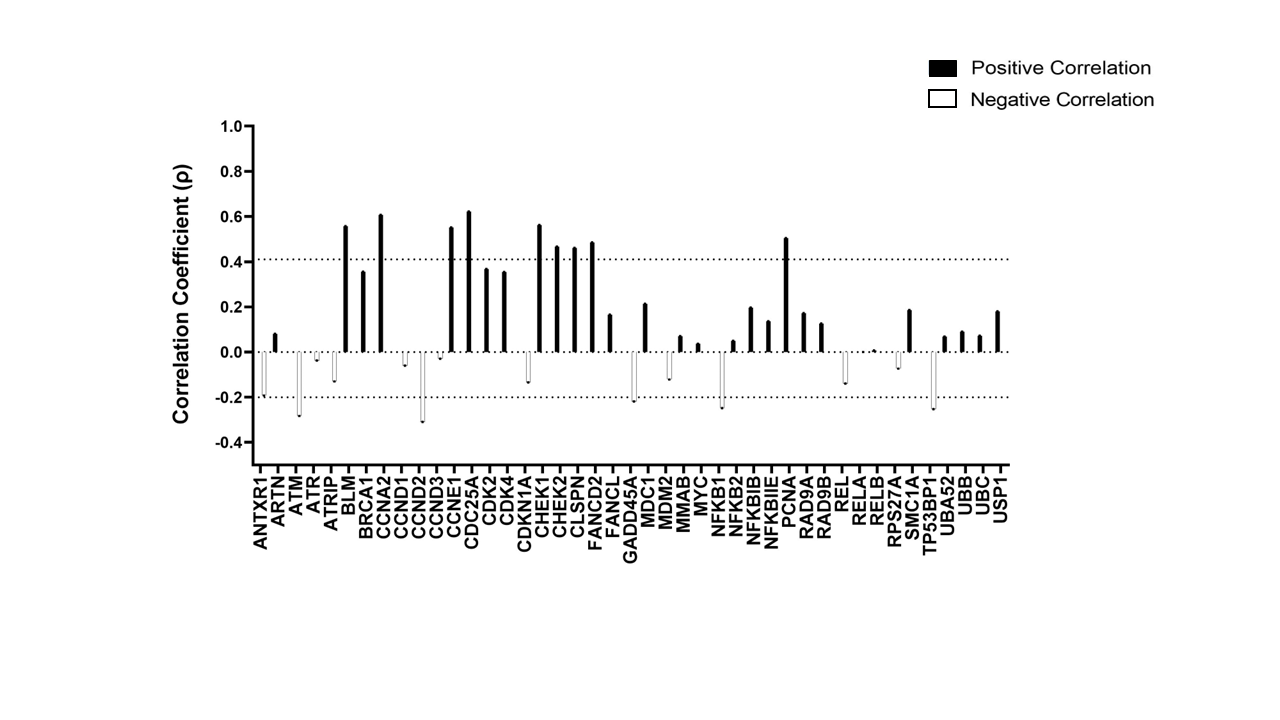

Supplement: S6 Fig — Significant Spearman correlations (p ≤ 0.05) are shown for positive (n = 28) and negative (n = 15) coefficients. Stronger correlations extend above or below two standard deviations that are denoted by horizontal asymptotes at 0.41 and -0.20. (TIF) [file pone.0293128.s006.tif]

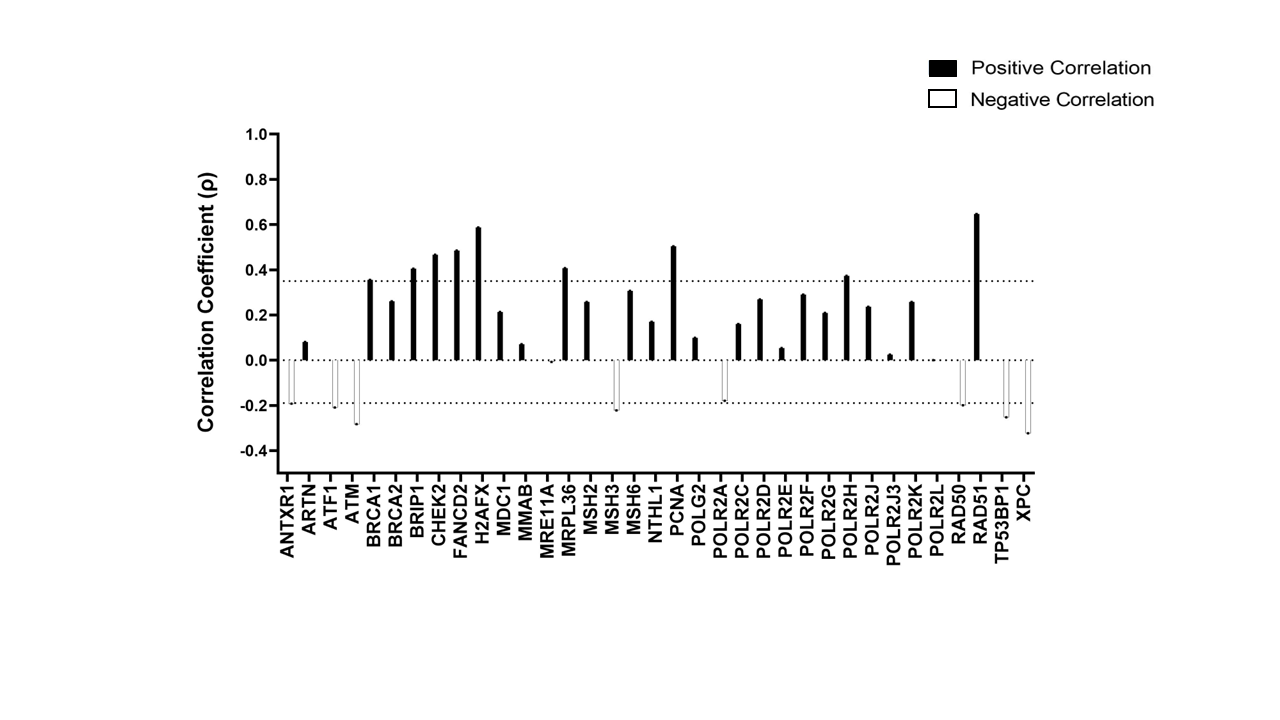

Supplement: S7 Fig — Significant Spearman correlations (p ≤ 0.05) are shown for positive (n = 26) and negative (n = 9) coefficients. Stronger correlations extend above or below two standard deviations that are denoted by horizontal asymptotes at 0.35 and -0.19. (TIF) [file pone.0293128.s007.tif]
