# Supplementary material for: TK1 expression influences pathogenicity by cell cycle progression, cellular migration, and cellular survival in HCC 1806 breast cancer cells
Source: PLoS One. 2023 Nov 30;18(11):e0293128. doi: 10.1371/journal.pone.0293128 (PMC10688958; doi:10.1371/journal.pone.0293128)
Supplement: S1 Raw images — This file is submitted as a PDF. (PDF) [file pone.0293128.s008.pdf]

Fig 2. Unedited Western Blot Images

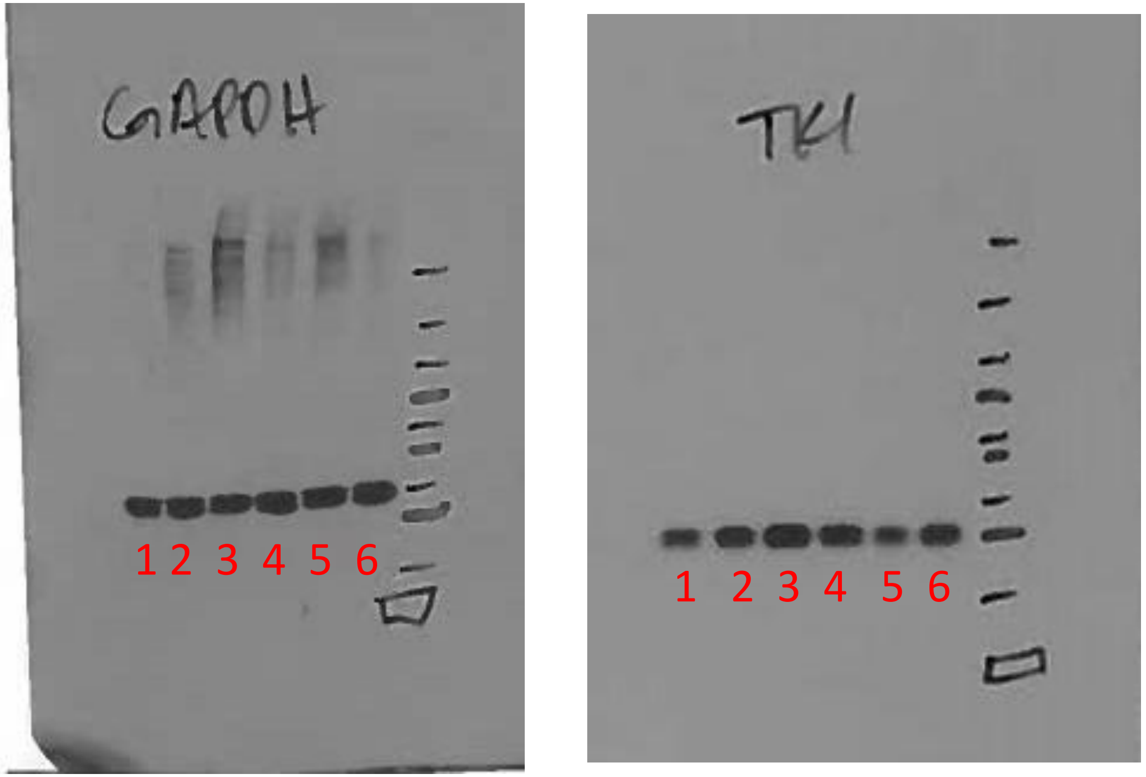

Fig 2: Unedited Western Blot images representative for breast cancer cell lines TK1 levels. For both the GAPDH and TK1 samples, the lanes are as follows: **1)** MCF7 **2)** MDA-MB-231 **3)** T47D **4)** HCC 1806 **5)** HCC 1937 **6)** JIMT-1. The GAPDH and TK1 samples were run on the same experimental gel. Several samples were analyzed on that day. For convenience and improved image quality, the samples are shown side by side in this view. It should be noted that in figure 2 of the paper, rows 4,5, and 6 were rearranged.

Fig 3. Unedited Western Blot Images

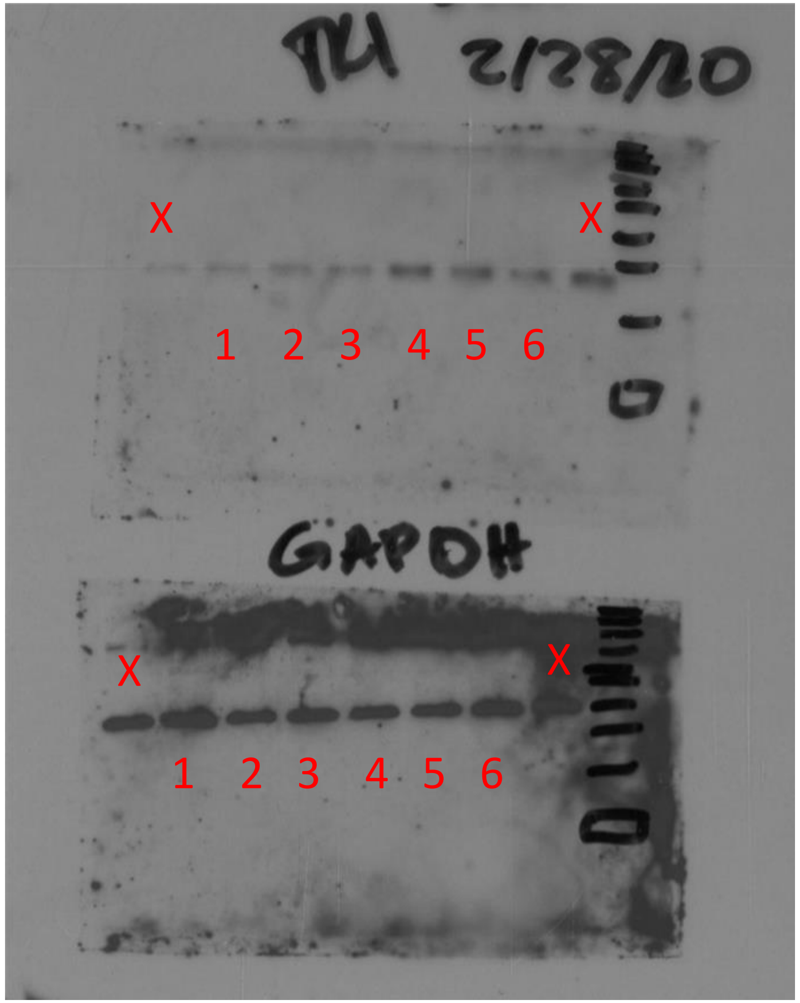

Fig 3: Unedited Western Blot images representing TK1 levels in HCC 1806 and L133 samples. Lanes 1-6 annotated in red in the image are those shown in figure 3 of the paper. Lanes 1-3 contain cell lysate from HCC 1806 cells and lanes 4-6 contain cell lysate from L133 cells.
